# Navigating the Gray Zone: AI Decision Support to Identify Aortic Stenosis Severity

**DOI:** 10.1016/j.jacadv.2024.101178

**Published:** 2024-09-25

**Authors:** Ashish Sarraju, David Ouyang

**Affiliations:** a Department of Cardiovascular Medicine and Cleveland Clinic Coordinating Center for Clinical Research, Cleveland Clinic Foundation, Cleveland, Ohio, USA; b Department of Cardiology and Division of Artificial Intelligence in Medicine, Cedars-Sinai Medical Center, Los Angeles, California, USA

**Keywords:** aortic stenosis, artificial intelligence, decision support, echocardiography

**Figure:**
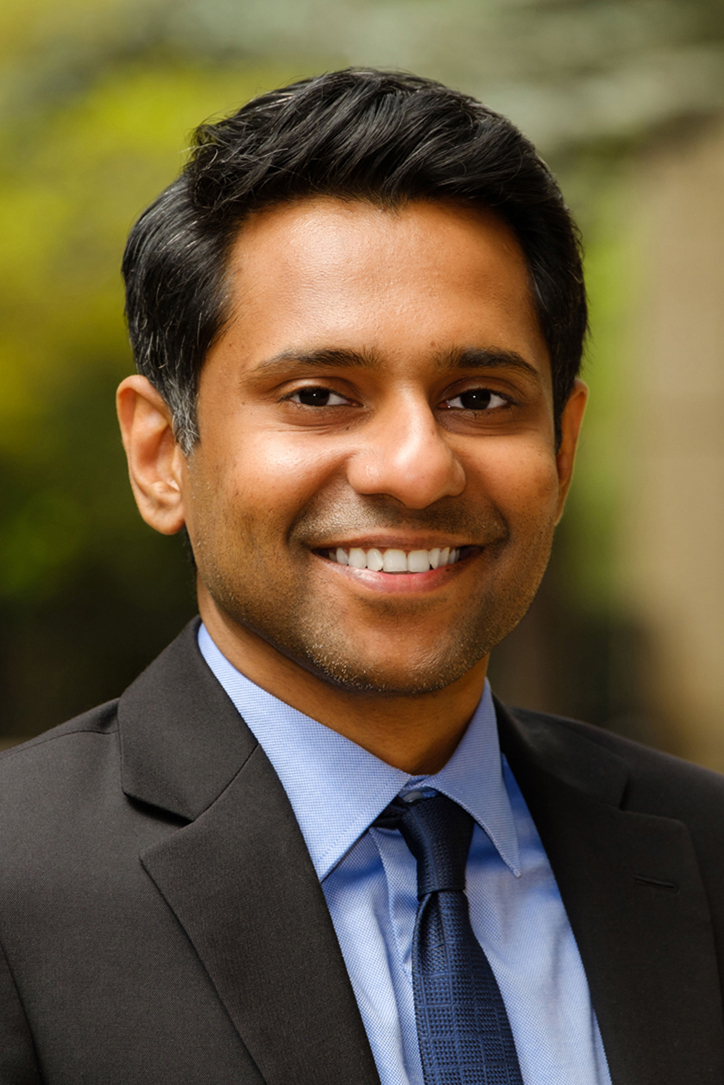

Diagnostic pathways within cardiology carry immense potential to benefit from artificial intelligence (AI)-enabled technologies.^1^ AI can track beat-to-beat ejection fraction, identify cardiac amyloidosis, and automate error-prone tedious measurements from images.^1^^,^^2^ As AI research for cardiovascular diagnosis gathers unprecedented momentum, a key question prevails: what cardiovascular conditions, or “use cases,” are ideal for the application of AI-based decision support? We postulate that conditions that are life- or quality of life-threatening and have proven therapies but suffer from diagnostic uncertainty, risk underestimation, and consequent significant practice variation may be well-suited to develop and study AI-enabled decision support to optimize diagnosis, risk assessment, and potential therapy allocation. Severe aortic stenosis (AS) is one such potential condition.

Symptomatic, severe AS is a high-risk valvular disease that requires aortic valve replacement (AVR) to improve symptoms and outcomes, including mortality. Transcatheter AVR options may be available even if surgical AVR is high risk. Therefore, identifying severe AS for timely and appropriate AVR referral has increasing importance in modern cardiology practice. Echocardiographic (echo) diagnosis—the standard modality—relies on a combination of directly measured variables including aortic valve velocities, transaortic gradients, and left ventricular outflow tract dimensions; and subsequent parameters calculated from the measured variables, including aortic valve area and the dimensionless valve index. Discordant parameters are not uncommon, which may lead to consternation regarding the presence of “true” severe AS—and the appropriate timing of referral for AVR. Suboptimal echo data due to technical limitations or variation in user-dependent measurements (such as left ventricular outflow tract dimension) may further add subjectivity to the evaluation of AS despite clear thresholds in guidelines. Diagnostic uncertainty may be exacerbated in patients with subtle or limited symptoms or concomitant disease that can be alternative etiologies for symptoms, such as cardiomyopathy, obstructive airway disease, or morbid obesity.

In this issue of *JACC: Advances*, Strom et al^3^ report the results of an external validation study of an AI decision support algorithm (AI-DSA) to identify the risk profiles and phenotypes of severe AS. This AI-DSA was originally trained on over 1 million echocardiograms from 631,824 individuals from Australia. In the present study, the study cohort had 31,141 Medicare beneficiaries, of whom over 80% were White, with approximately 52% women, and a mean age of approximately 77 years. Probability thresholds were established for severe AS (which the authors label F1) and moderate AS (which the authors label as F2) by AI-DSA. Four patient groups were defined based on the probability of severe AS as identified by AI-DSA and the presence of severe AS by traditional guidelines: group 1, with severe AS by both AI-DSA (probability above the F1 threshold) and by guidelines; group 2, with severe AS by AI-DSA but not by guidelines; group 3, with moderate AS by AI-DSA (probability below F1 but above the F2 threshold); and group 4, with a low probability of severe AS by AI-DSA (below the F2 threshold). The authors found strong overall performance of the AI-DSA, as defined by an area under the receiving operating characteristic curve of 0.986, for the identification of AVA <1 cm^2^. Groups 1 to 3 had significantly higher adjusted mortality risk as compared with group 4 by Cox proportional hazards. Five-year mortality was highest in group 2, in which patients met the AI-DSA threshold but not traditional guideline criteria for severe AS, followed by group 1, in which AI-DSA and guidelines were concordant for severe AS.

This study by Strom et al complements the existing literature involving recognizing and risk stratifying AS. By studying the AI-DSA in an external validation health system in a different continent as compared with the initial training and internal validation data set, the authors highlight the robustness of their approach in risk stratifying AS, even among patients that do not meet the classic definitions of AS. However, the present cohort was restricted to predominantly White Medicare enrollees, presumably mainly with calcific disease, therefore performance with less common etiologies such as bicuspid AS and in diverse populations is not specifically addressed in this study. By using report measurements as input rather than the images themselves, the AI-DSA is dependent on accurate clinician-generated measurements for analysis. Inaccurate over- or under-measurement by clinicians may change the risk stratification of patients.

The authors identified a morbid AS phenotype as detected by AI-DSA that did not meet guideline criteria for severe AS but demonstrated a mortality risk comparable to those who met both AI-DSA thresholds and guideline criteria for severe AS. Because of the exclusion of this group from severe AS by guidelines, this group underwent fewer eventual AVRs, which highlight potential gaps in current understanding of AS. With these data the authors raise the compelling hypothesis of whether a probabilistic approach to identifying severe AS by echo (that is, defining AS severity based on reaching a threshold of probability of future risk) can augment the traditional “categorical” approach (that is, defining severe AS based on the binary presence versus absence of a specific echo measurement) to better identify benefit from treatment, especially for a condition that is generally progressive and potentially fatal if untreated. Validated AI technologies have the potential to augment probabilistic diagnostic pathways in a scalable manner.

Several questions remain for the future. Can AI approaches consistently detect patients with AS who will benefit from AVR despite “slipping through the cracks” of traditional severe AS diagnostic criteria? Can AI-enabled approaches homogenize practice variation regarding severe AS diagnosis and AVR referral in a scalable and equitable manner that improves AS outcomes? Prospective study is required to definitively address these considerations. Ultimately, to determine whether AI decision support will be beneficial in routine care, we may need a workflow-embedded study of not only clinical outcomes but also clinician time burden, cost-effectiveness, and automation bias as a risk. For now, the present study shows the promise of AI-based decision support to sharpen the sometimes-hazy echo definitions of severe AS in a prognostically relevant manner.

## Funding support and author disclosures

Dr Ouyang has received support from the 10.13039/100000002National Institutes of Health (10.13039/100000002NIH; 10.13039/100000050NHLBI R00HL157421) and Alexion, and consulting or honoraria for lectures from EchoIQ, Ultromics, 10.13039/100004319Pfizer, InVision, the 10.13039/100010153Korean Society of Echo, and the 10.13039/100018112Japanese Society of Echo. Dr Sarraju has reported that he has no relationships relevant to the contents of this paper to disclose.
